# MadgwickFall-Net: A Lightweight Dual-Frame Feature Fusion Network for Pre-Impact Fall Detection Using Wearable IMUs

**DOI:** 10.3390/bioengineering13050568

**Published:** 2026-05-16

**Authors:** Qijun Zhong, Jing Wang, Guiling Sun

**Affiliations:** 1 College of Electronic Information and Optical Engineering, Nankai University, Tianjin 300350, China; 2312622@mail.nankai.edu.cn (Q.Z.); 2413615@mail.nankai.edu.cn (J.W.); 2 Tianjin Key Laboratory of Optoelectronic Sensor and Sensing Network Technology, Tianjin 300350, China

**Keywords:** pre-impact fall detection, dual-frame feature fusion, madgwick algorithm, temporal convolutional network, inertial measurement unit

## Abstract

As global population aging intensifies, fall-related injuries among the elderly have become a critical public health concern. Existing fall detection methods based on wearable IMUs all extract features in the sensor’s body frame, failing to exploit the information embedded in sensor signals. Some higher-performing methods incorporate magnetometer-fused Euler angles to enrich features, but their dependence on specific hardware and fusion algorithms makes exact replication during deployment difficult. In contrast, the proposed MadgwickFall-Net relies on acceleration and angular velocity, and, to the best of our knowledge, for the first time introduces the Madgwick algorithm into fall detection to transform inertial signals into a gravity-aligned global coordinate system. A four-branch parallel architecture processes signals from both coordinate frames, fully exploiting the complementarity between dual-frame signals. Cross-validation on the KFall dataset using 5-fold subject-independent stratification demonstrates an F1-Score of 0.9824 and accuracy of 98.36%, specifically, four main evaluation indicators outperform all comparison models. With only 59.7 KB parameters, the model is suitable for edge device deployment. Rolling inference experiments demonstrate a median pre-impact lead time of 390 ms. MadgwickFall-Net offers a practical and deployable solution for real-world wearable fall detection systems, demonstrating strong potential for protecting elderly individuals in daily life scenarios.

## 1. Introduction

As global population aging continues to intensify, the health and safety of the elderly have become critical issues for social development and public welfare. Falls are one of the most common and severe health problems affecting older adults, with approximately 26.5% of elderly individuals experiencing at least one fall event annually [1]. Data shows that in 2021, the number of fall-related deaths among people aged ≥55 worldwide reached approximately 637,000 [2], and high-income countries spend about 1% of their total health budgets on fall-related medical expenses [3]. This phenomenon not only inflicts severe physical and psychological trauma on the elderly but also imposes a heavy socioeconomic and caregiving burden on families and healthcare systems. Biomechanical studies indicate that the core mechanism of fall-related injuries lies in the enormous impact force generated when the human body collides with the ground [4]. Therefore, developing a practical pre-impact fall detection system is of great significance for reducing fall risks and improving safety protection levels for the elderly.

Existing fall detection technologies can be broadly categorized into three types based on sensor modalities [5]: wearable sensors [6], environmental sensors [7,8], and vision-based sensors [9]. Environmental and vision-based sensor methods require fixed installation of equipment in specific scenarios, which not only creates detection blind spots but also raises concerns about user privacy leakage [10,11]. With the rapid development of micro-electromechanical systems technology, researchers have successfully integrated inertial measurement units (IMUs) into small wearable devices for fall detection [5,12,13,14]. Wearable sensor-based fall detection methods offer significant advantages such as low deployment cost, strong environmental adaptability, and effective user privacy protection. Consequently, lightweight neural networks deployable on wearable devices have become an active and highly promising research direction in the field of fall detection [12,15]. Research has demonstrated that observable kinematic changes in the human body emerge hundreds of milliseconds before ground impact [16], providing biomechanical justification for the feasibility of capturing fall-related motion features via inertial sensors prior to impact and thereby enabling pre-impact fall detection.

**Figure 1:**
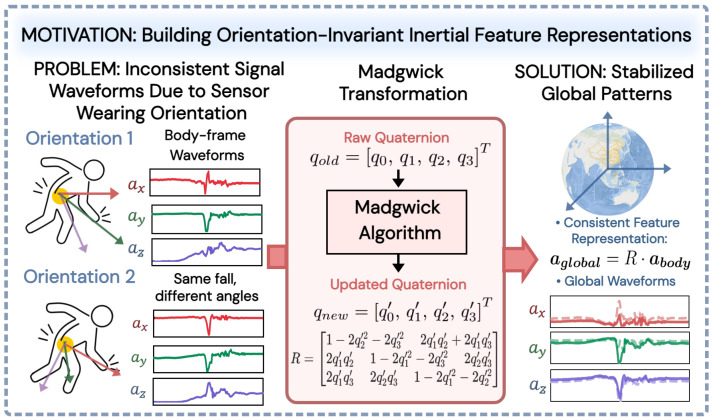
Signal waveform inconsistency caused by wearing orientation in the body frame and its correction via the Madgwick transformation to stable global representations.

However, most existing neural networks deployed on wearable devices directly process raw sensor signals in the body frame [6,12,17], implicitly assuming that the wearing orientation is consistent across all subjects [18]. In practical applications, there are individual differences in the wearing orientation of sensors among different subjects, and the same fall motion exhibits significantly different waveform characteristics in the body frame due to varying wearing orientation [18,19], as shown in Figure 1, posing challenges for feature extraction by neural networks. From a biomechanical perspective, key impact parameters during a fall (such as the vertical impact velocity and acceleration of the hip) can only be physically consistently described when calculated in a gravity-aligned global frame [20]. Therefore, extracting inertial signal features in the global coordinate system can preserve the orientation-independent physical description of fall dynamics, serving as an effective approach to overcome the limitations of local coordinate system representations. In addition, some methods use Euler angles derived from extended Kalman filter fusion of accelerometers and magnetometers as input features [21,22], which are difficult to fully replicate during deployment, leading to train-deployment mismatches and limiting the practical deployability of the models.

Despite the long-standing existence of the above issues in the field of fall detection, existing work has not yet systematically introduced mature attitude estimation algorithms into this scenario to construct gravity-aligned inertial feature representations that are invariant to wearing orientation through coordinate transformation. Meanwhile, the complementarity of sensor signals under the local and global coordinate systems has never been explicitly modeled and exploited.

Based on the above observations, this paper introduces the Madgwick algorithm [23] to preprocess the raw inertial signals. This algorithm represents the attitude using quaternions, iteratively estimates gyroscope measurement errors via gradient descent, compensates for angular velocity integration, and outputs stable attitude quaternions. Using these quaternions as rotation operators, the acceleration and angular velocity signals in the body frame are projected into a gravity-aligned global frame, yielding motion representations independent of the sensor’s wearing orientation.

Meanwhile, this paper proposes MadgwickFall-Net, a lightweight four-branch convolutional neural network. As the first fall detection method that performs parallel feature extraction and fusion on sensor signals in both the local and global coordinate systems, it introduces a coordinate transformation module in the data preprocessing stage to convert signals from the body coordinate system into the gravity-aligned global coordinate system. Signals under the dual coordinate systems are then fed into separate independent network branches in parallel, enabling the model to simultaneously capture the complementary representations of local dynamic features and global motion features.

Ablation experiments show that the complete four-branch model integrating dual coordinate systems achieves optimal detection performance (F1 = 0.9824, Recall = 0.9876). Compared with the two-branch configuration using only the global frame (Madgwick output), the F1-score improves by 0.0101, and compared with the two-branch configuration using only the body frame (without Madgwick), the F1-score improves by 0.0164, verifying the effectiveness of the parallel modeling strategy based on dual coordinate systems.

The main contributions of this paper are summarized as follows:

1. To the best of our knowledge, this is the first work that introduces the Madgwick attitude fusion algorithm into the field of pre-impact fall detection, which transforms inertial signals into the gravity-aligned global coordinate system and constructs inertial feature representations at the feature level that are invariant to sensor wearing orientations. Furthermore, the optimal gain configuration is determined via systematic ablation experiments on the β parameter.
2. To address the problem of insufficient feature extraction in the body frame in existing methods, a lightweight four-branch parallel neural network, MadgwickFall-Net, is proposed. By processing acceleration and angular velocity signals from dual-frame in parallel, the complementary attitude kinematic features in inertial signals are fully exploited.
3. A reverse sliding window sampling strategy for pre-impact detection is proposed, which samples forward with the impact cutoff point as the anchor point to maximize the retention of key pre-fall information near the cutoff point, and effectively alleviates the class imbalance problem combined with data augmentation.

## 2. Related Work

### 2.1. IMU-Based Fall Detection Methods

As a universal motion sensing platform, IMUs have been widely applied in multiple elderly health monitoring tasks, including fall detection, balance function assessment [24], and gait independence evaluation [25]. Research on IMU-based fall detection has gone through three development stages: threshold-based methods, traditional machine learning, and deep learning [26].

With the rise of deep learning, CNN has become the mainstream choice due to its end-to-end feature learning capability. Yu et al. [15] proposed TinyCNN, which achieves efficient modeling of temporal dependencies in inertial signals through two-stage decoupled feature extraction with temporal and spatial convolution, combined with a global average pooling layer. To further capture temporal dependencies, some studies have introduced LSTM: Hu et al. [27] constructed a hybrid CNN-LSTM model consisting of a two-layer feature extractor and a single-layer LSTM, which extracts spatial features of wrist-worn IMU signals via CNN and inputs them into LSTM to model temporal dependencies, realizing effective detection of involuntary falls; Kiran et al. [28] adopted a CNN-BiGRU integrated architecture, which enhances pre-impact fall detection capability through multi-scale convolution and collaborative modeling of bidirectional gated recurrent units.

Recently, research has shifted to more complex architectures to pursue performance upper limits. Choi et al. [29] classified near-falls as a third category, proposing a DAG-CNN architecture that fuses multi-level convolutional features through directed acyclic graph structures, but this architecture takes crafted manually statistical features as input and fails to achieve end-to-end learning from raw signals; Al-qaness et al. [30] proposed a PCNN-Transformer architecture, which arranges multiple residual CNN blocks and Transformer encoders in parallel, extracts local convolutional features through a residual projection mechanism, models global temporal dependencies using a multi-head self-attention mechanism, and finally fuses the two features through concatenation to complete classification; Bhatti et al. [31] constructed a hybrid CNN-LSTM-Attention architecture by introducing an attention mechanism, expanding the detection scope from pre-impact to post-impact and transition phases; Zafar and Zafar [32] introduced the self-attention mechanism of Transformer encoders into fall detection, improving activity recognition capability by modeling local and global temporal dependencies.

All the above methods face common bottlenecks. First, existing methods generally extract features directly in the sensor’s local coordinate system [27,28,29,30,31,32], failing to fully exploit the physically consistent information contained in the gravity-aligned global coordinate system, and the learned feature representations are strongly coupled with the sensor measurement coordinate system [19]. Second, some recent complex architectures have a large number of parameters [30,31], making them difficult to deploy directly on embedded wearable devices, which restricts the practical implementation of fall detection systems.

To this end, this paper proposes to use the output of the attitude estimation algorithm as the basis for coordinate transformation and convert the raw inertial signals from the sensor’s body frame to the global frame before feature extraction. Integrated with a lightweight 1D-CNN backbone, it achieves physically consistent global feature learning with embedded-friendly computational complexity.

### 2.2. Methods for Achieving Orientation Invariance in IMU Sensors

Research on achieving orientation invariance for IMU sensors mainly follows two paths: coordinate normalization and data augmentation. In terms of coordinate normalization, Nouriani et al. [33] designed a nonlinear high-gain observer based on Lyapunov analysis, which jointly estimates the roll and pitch angles of the sensor in the global frame using the accelerometer and gyroscope measurements from a single IMU, thereby eliminating the impact of wearing rotation. Son and Choi [34] proposed a two-stage coordinate transformation scheme: first, the accelerometer is used to estimate the pitch and roll angles to eliminate device tilt, and then the gyroscope yaw angle is utilized to align vectors to an application-specific reference coordinate system, achieving orientation invariance compensation for all three-axis rotations. In terms of data augmentation, Um et al. [35] mitigated orientation variability at the data level through rotation augmentation to simulate different wearing orientations; Oishi et al. [36] further constrained rotations within physically reasonable ranges and introduced position offsets, making the augmented data more consistent with real-world wearing variations. However, the data augmentation path has a common limitation: its intervention only functions during the training phase, and the model still directly faces raw signals in the body frame during inference, leaving the orientation invariance problem fundamentally unresolved at the feature level.

In contrast, the lightweight attitude estimation algorithm fusing accelerometers and gyroscopes proposed by Madgwick et al. [23] retains gravity-aligned absolute orientation information with low computational overhead, providing a lightweight and feasible approach for constructing global coordinate system representations at the feature level.

However, existing work has insufficiently utilized this approach: Lin et al. [37] only used the pitch and roll angles output by the Madgwick algorithm as threshold criteria for state machines; He et al. [38] transformed accelerometer signals into the global frame using the quaternions output by the Madgwick algorithm, generating only global acceleration as a fusion component and concatenating it with the original 6D signals to form 9D input; Mohammed et al. [39] only estimated the rotational attitudes of the thoracic, hip, and knee joints through quaternion integration using IMU gyroscope data, calculated joint angles based on T-pose for fall prediction, and did not fuse accelerometer information for attitude correction.

In summary, coordinate normalization methods either rely on high-computational-overhead solutions such as high-gain observers [33] or require additional hardware support from magnetometers and external positioning systems [34], making it difficult to deploy independently on standard wearable IMUs; data augmentation only acts during the training phase and cannot fundamentally eliminate orientation dependence during inference; existing quaternion-based solutions also only utilize the Madgwick algorithm partially. None of the three paths have systematically constructed an orientation invariance mechanism at the feature level in the fall detection scenario.

## 3. Methods

This section introduces DualFrame-PFD, a training framework for pre-impact fall detection models based on dual-coordinate system feature fusion. It covers the data preprocessing pipeline and the architectural design of the neural network component, MadgwickFall-Net.

### 3.1. Overall Framework of the Method

**Figure 2:**
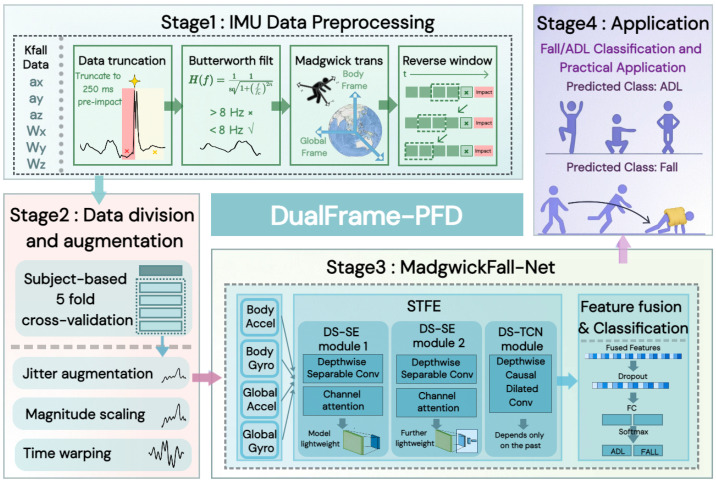
Overview of the DualFrame-PFD framework, comprising IMU data preprocessing, data division and augmentation, MadgwickFall-Net architecture, with binary classification output of ADL or Fall.

Figure 2 details the complete workflow of DualFrame-PFD, which consists of three core modules: IMU data preprocessing, data division and augmentation, and the MadgwickFall-Net network architecture.

- Data Input Stage: The KFall dataset [40] provides 6-channel inertial signals, including triaxial acceleration and triaxial angular velocity, and this method uses only these 6-channel signals as the raw input.
- IMU Data Preprocessing Stage: The raw signals sequentially undergo four steps: data truncation, Butterworth low-pass filtering, Madgwick algorithm, and reverse sliding window sampling, ultimately outputting acceleration and angular velocity signals in both the original and global frames, resulting in 4 data streams as parallel inputs to the four-branch network of MadgwickFall-Net. Specifically, first, based on the annotation of the fall impact time in the dataset, all sensor data after 250 ms before the impact is removed. The 250 ms cutoff is set with reference to the average response time of approximately 200 ms for wearable airbags from trigger to full deployment [41]. Additional inference computation margin is reserved on this basis so that the trained model can complete detection and trigger an alarm before 250 ms prior to impact, providing a sufficient time window for the deployment of the protection device. Second, a 4th-order Butterworth low-pass filter (cutoff frequency 8 Hz) is applied to the truncated data to filter out high-frequency noise and obtain smooth raw acceleration and angular velocity signals. On this basis, the Madgwick algorithm is used to transform the acceleration and angular velocity from the body frame to the global frame, eliminating the influence of sensor attitude changes on motion features. Finally, for fall data, we take 250 ms before the fall impact as the starting point and adopt a reverse sliding window sampling strategy (window length 875 ms, overlap rate 60%), then input the data to the model for training in chronological order; for activities of daily living (ADL) data, a conventional forward sliding window sampling strategy is used for sampling.
- Data Division and Augmentation Stage: The preprocessed data is divided into 5-fold cross-validation splits by subject, strictly ensuring that data from the same subject does not appear in both the training and test sets simultaneously, thereby effectively improving the model’s generalization ability to unseen subjects. To ensure the objectivity of evaluation, the training set is used to train model parameters, while the test set is fully sealed during the training process and used only once in the final evaluation. To address the class imbalance between fall and ADL samples, this method applies three data augmentation methods—jitter, scaling, and time warping—to the fall data in the training set, achieving a 7-fold data augmentation, which brings the ratio of fall samples to ADL samples to approximately 1:1. After the above processing, MadgwickFall-Net is trained on the balanced data, and finally outputs binary classification results: ADL or Fall.

Three data augmentation techniques are exclusively applied to fall samples in the training set. All parameters are fixed for reproducibility:

- Jitter: Additive Gaussian noise with σ=0.04;
- Scaling: Random scaling factor sampled from N(1.0,0.10);
- Time Warping: Spline warping with σ=0.15 and 4 knots;

### 3.2. Reverse Sliding Window Sampling Strategy

Existing literature mostly adopts forward sliding window sampling for fall data [21,42], where windows slide sequentially from the start to the end of the fall sequence, terminating at the impact moment [42]. However, under the forward sliding window strategy, data segments shorter than a full window length before the cutoff point cannot form valid samples and are directly discarded, resulting in the waste of critical motion information near the cutoff point.

**Figure 3:**
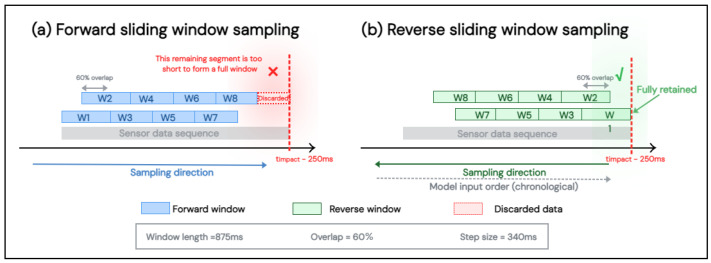
Comparison of sliding window sampling strategies. (**a**) Forward sampling: windows slide toward the cutoff point, leaving a terminal segment discarded. (**b**) Reverse sampling: windows are anchored at the cutoff point and slide backward, ensuring full retention of pre-impact data. Window length = 875 ms, overlap = 60%, step size = 340 ms.

To address this issue, this paper proposes a reverse sliding window sampling strategy: taking the cutoff point as the sampling starting point, windows are generated by sliding forward sequentially, ensuring that the end of each window is aligned with or close to the cutoff point. This maximizes the retention of key motion information near the cutoff point and improves the data utilization rate in the pre-impact phase, as illustrated in Figure 3. The reverse sliding window strategy is presented in Algorithm 1.

It is worth noting that the difference in window anchoring methods between fall and activities of daily living (ADL) data stems from the fundamental difference in their temporal structures. ADL has no specific critical moment, and forward sliding windows can uniformly cover the entire activity period; in contrast, the discriminative features of falls are concentrated near the impact cutoff point, and reverse anchoring maximizes the retention of information in this critical region.

The difference between the two strategies lies only in the window anchoring position, and the internal data of each window fed into the network remains in the original chronological order. In addition, windows sampled from fall trials via the reverse sliding window are assigned labels of fall (1) or ADL (0) based on the overlap ratio between their time intervals and the annotated fall phases. Both labels are distributed within a single trial, and the label assignment is independent of the sampling position of the window, but determined by the actual time of the motion event.
**Algorithm** **1** Reverse Sliding Window Sampling for Pre-Impact Fall Detection**Require:** Fall IMU sequence *S*, impact timestamp $t_{impact}$, sampling rate $f_{s}=100$ Hz**Require:** Window length L=875 ms, overlap rate 60%, pre-impact margin $T_{margin}=250$ ms**Ensure:** Set of valid pre-impact windows *W* 1:Compute cutoff time: $t_{cut}=t_{impact}-T_{margin}$ 2:Truncate sequence: $S_{trunc}=\mathrm{data}\;\mathrm{of}\;S\;\mathrm{before}\;t_{cut}$ 3:Convert window length to samples: $N_{win}=\left\lfloor L\cdot f_{s}/1000\right\rfloor =87$ 4:Compute step size: $step=\lfloor N_{win}\cdot \left(1-0.6\right)\rfloor =34$ 5:Initialize window set W←∅ 6:Set current end position $pos\leftarrow \mathrm{length}(S_{trunc})$ 7:**while** $pos-N_{win}\geq 0$ **do** 8:     Extract window: $w=S_{trunc}\left[pos-N_{win}:pos\right]$ 9:     Add *w* to *W*10:   Move backward: pos←pos−step11:**end while**12:**return** *W*

Notably, the reverse sliding window strategy is only employed during the offline dataset construction phase. In the real-time inference stage, the system adopts a conventional forward-sliding buffer with a step size of 340 ms, feeding the most recently accumulated 875 ms window into the model at each inference. A quantitative performance comparison between the forward and reverse sliding window strategies, as well as a discussion of the potential train-deployment mismatch problem, is presented in Section 4.4.2.

### 3.3. Coordinate Transformation Based on Madgwick Algorithm

Throughout this paper, motion is described with respect to two coordinate systems: the gravity-aligned global frame and the sensor body frame. The global frame is defined as a right-handed coordinate system in which the Z-axis points vertically upward (i.e., opposite to the direction of gravity), the X-axis points forward along the subject’s facing direction, and the Y-axis points toward the left side of the human body. The body frame is a coordinate system rigidly attached to the sensor housing, whose three axes are aligned with the physical axes of the sensor package and co-rotate with changes in wearing orientation.

The Madgwick algorithm plays a central role in the preprocessing pipeline: it fuses accelerometer and gyroscope measurements to estimate the sensor attitude quaternion q in real time, constructs the corresponding rotation matrix, and projects the signals from the sensor’s body frame into a gravity-aligned global coordinate frame.

The sensor attitude quaternion is defined as:

(1) $$q={\left[\begin{array}{cccc} q_{0} & q_{1} & q_{2} & q_{3} \end{array}\right]}^{⊤},\;∥q∥=1$$

where $q_{0}$ is the scalar component, and $q_{1},q_{2},q_{3}$ are the vector components. The quaternion is initialized as $q_{0}={\left[1,0,0,0\right]}^{⊤}$, corresponding to the state where the body frame is aligned with the global frame. The attitude estimation is divided into four stages: gyroscope-driven prediction, accelerometer-based gradient descent correction, quaternion integration with normalized update, and coordinate transformation.

Stage 1: Gyroscope-driven prediction. Let $\omega ={\left[\omega_{x},\omega_{y},\omega_{z}\right]}^{⊤}$ denote the triaxial angular velocity measured in the sensor’s body frame (unit: $\mathrm{rad}\cdot s^{-1}$). The rate of change in the attitude quaternion induced by angular rotation is calculated as follows:

(2) $${\dot{q}}_{\omega }=\frac{1}{2}q⊗\left[\begin{array}{c} 0\\ \omega_{x}\\ \omega_{y}\\ \omega_{z} \end{array}\right]$$

where ⊗ denotes the Hamilton quaternion multiplication.

Stage 2: Accelerometer-based gradient descent correction. First, the raw accelerometer measurement $a={\left[a_{x},a_{y},a_{z}\right]}^{⊤}$ is normalized to obtain a unit vector that carries only directional information:

(3) $$\hat{a}=\frac{a}{∥a∥}={\left[\begin{array}{ccc} \frac{a_{x}}{∥a∥} & \frac{a_{y}}{∥a∥} & \frac{a_{z}}{∥a∥} \end{array}\right]}^{⊤}$$

In the global frame, the gravity reference direction is fixed as $g={\left[0,0,1\right]}^{⊤}$. The rotation matrix corresponding to the current quaternion q is used to transform g into the body frame, yielding the predicted gravity direction $\hat{g}\left(q\right)$:

(4) $$\hat{g}\left(q\right)=R^{⊤}{\left[0,0,1\right]}^{⊤}$$

which corresponds to the third column of $R^{⊤}$ (equivalently, the third row of R). The deviation between the predicted gravity direction and the measured direction $\hat{a}$ is defined as the attitude deviation objective function:

(5) $$f\left(q,\hat{a}\right)=\hat{g}\left(q\right)-\hat{a}=\left[\begin{array}{c} 2\left(q_{1}q_{3}-q_{0}q_{2}\right)-{\hat{a}}_{x}\\ 2\left(q_{0}q_{1}+q_{2}q_{3}\right)-{\hat{a}}_{y}\\ 2\left(\frac{1}{2}-q_{1}^{2}-q_{2}^{2}\right)-{\hat{a}}_{z} \end{array}\right]$$

To minimize this objective function via gradient descent, the partial derivatives of f with respect to each quaternion component $q_{0},q_{1},q_{2},q_{3}$ are computed, yielding the 3×4 Jacobian matrix:

(6) $$J=\frac{\partial f}{\partial q}=\left[\begin{array}{cccc} -2q_{2} & 2q_{3} & -2q_{0} & 2q_{1}\\ 2q_{1} & 2q_{0} & 2q_{3} & 2q_{2}\\ 0 & -4q_{1} & -4q_{2} & 0 \end{array}\right]$$

The gradient of the objective function and its normalized form are then calculated as:

(7) $$\nabla f=J^{⊤}f\left(q,\hat{a}\right),\;\hat{\nabla }f=\frac{\nabla f}{∥\nabla f∥}$$

The gyroscope-driven prediction is fused with the accelerometer-based correction along the normalized gradient descent direction, yielding the composite quaternion rate of change:

(8) $$\dot{q}={\dot{q}}_{\omega }-\beta \hat{\nabla }f$$

where β is a gain parameter that governs the correction strength of the accelerometer on gyroscope drift. An excessively large β renders the system overly sensitive to accelerometer noise, while an excessively small β leads to insufficient drift suppression. Based on the ablation experiment results presented in Section 4.3.3, β is set to 0.1 in this paper.

When the sensor is in an approximate free-fall state, the acceleration magnitude approaches zero and can no longer serve as a reliable gravity reference. To handle this, a threshold of ε=0.1g is introduced; the gradient descent correction step is applied only when the measured acceleration magnitude exceeds this threshold.

Stage 3: Quaternion integration and normalization. The quaternion at the next time step is obtained via first-order forward Euler integration, followed by normalization to maintain unit length:

(9) $$q_{t+1}=q_{t}+\dot{q}\cdot \Delta t,\;q_{t+1}\leftarrow \frac{q_{t+1}}{∥q_{t+1}∥}$$

where Δt=0.01 s is determined by the 100 Hz sampling rate of the KFall dataset.

Stage 4: Coordinate transformation. After updating the quaternion, the corresponding 3×3 rotation matrix is constructed as:

(10) $$R=\left[\begin{array}{ccc} 1-2(q_{2}^{2}+q_{3}^{2}) & 2(q_{1}q_{2}-q_{0}q_{3}) & 2(q_{1}q_{3}+q_{0}q_{2})\\ 2(q_{1}q_{2}+q_{0}q_{3}) & 1-2(q_{1}^{2}+q_{3}^{2}) & 2(q_{2}q_{3}-q_{0}q_{1})\\ 2(q_{1}q_{3}-q_{0}q_{2}) & 2(q_{2}q_{3}+q_{0}q_{1}) & 1-2(q_{1}^{2}+q_{2}^{2}) \end{array}\right]$$

Through the rotation matrix R, the inertial signals in the sensor’s body coordinate system are projected onto the global gravity coordinate system:

(11) $$a_{\mathrm{global}}=R\cdot a_{\mathrm{body}},\;\omega_{\mathrm{global}}=R\cdot \omega_{\mathrm{body}}$$

thus yielding the acceleration and angular velocity representations in the global frame. The complete procedure of the above algorithm is shown in Algorithm 2.
**Algorithm** **2** Madgwick Attitude Estimation and Coordinate Transformation Algorithm**Require:** Raw acceleration a[t], raw angular velocity ω[t], t=0,1,…,N−1**Require:** Parameters: β=0.1, Δt=0.01 s**Ensure:** Global acceleration $a_{\mathrm{global}}[0$…N−1], global angular velocity $\omega_{\mathrm{global}}[0$…N−1] 1:Initialize q←[1,0,0,0] 2:**for** t=0 **to** N−1 **do** 3:   {1. Gyroscope integration} 4:   $\omega_{\mathrm{rad}}\leftarrow \deg2\mathrm{rad}\left(\omega \left[t\right]\right)$ 5:   ${\dot{q}}_{\omega }\leftarrow \frac{1}{2}\cdot q⊗\left[0,\omega_{\mathrm{rad}}\right]$ 6:   {2. Accelerometer correction} 7:   **if** ∥a[t]∥ >ε **then** 8:      $\hat{a}\leftarrow a\left[t\right]/∥a\left[t\right]∥$ 9:      $f\leftarrow \left[2\left(q_{1}q_{3}-q_{0}q_{2}\right)-{\hat{a}}_{x},\;2\left(q_{0}q_{1}+q_{2}q_{3}\right)-{\hat{a}}_{y},\;2\left(\frac{1}{2}-q_{1}^{2}-q_{2}^{2}\right)-{\hat{a}}_{z}\right]$10:     $J\leftarrow \frac{\partial f}{\partial q}$ {3×4 Jacobian matrix}11:     $\nabla f\leftarrow J^{T}\cdot f$12:     $\dot{q}\leftarrow {\dot{q}}_{\omega }-\beta \cdot \nabla f/∥\nabla f∥$ {Fusion}13:   **else**14:      $\dot{q}\leftarrow {\dot{q}}_{\omega }$ {Gyroscope only}15:   **end if**16:   {3. Integration + Normalization}17:   $q\leftarrow q+\dot{q}\cdot \Delta t$18:   q←q/∥q∥19:   {4. Coordinate transformation}20:   R← rotation_matrix (**q**) {3 × 3 rotation matrix}21:   $a_{\mathrm{global}}\left[t\right]\leftarrow R\cdot a\left[t\right]$22:   $\omega_{\mathrm{global}}\left[t\right]\leftarrow R\cdot \omega \left[t\right]$23:**end for**24:**return** $a_{\mathrm{global}}[0$…N−1], $\omega_{\mathrm{global}}[0$…N−1]

### 3.4. MadgwickFall-Net Network Architecture

**Figure 4:**
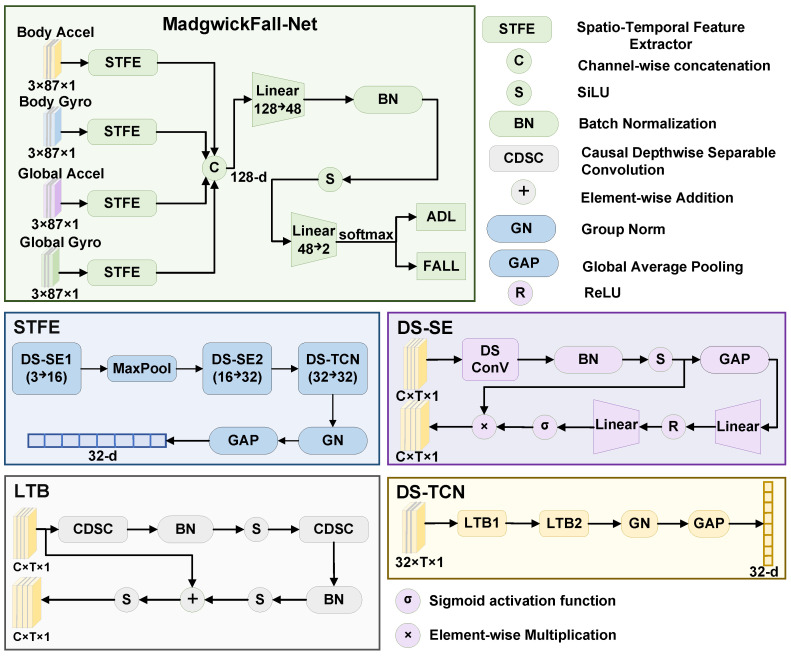
Overall architecture of MadgwickFall-Net, with four independent parallel STFEs (each comprising DS-SE modules, max-pooling, and a DS-TCN module with stacked LTBs) processing dual-frame inertial signals, followed by late-fusion concatenation and fully connected classification.

Based on the temporal dynamic characteristics of fall events, this paper proposes a lightweight neural network named MadgwickFall-Net that combines CNN and TCN. Its overall architecture, as shown in Figure 4, adopts a late fusion strategy: four parallel Spatio-Temporal Feature Extractors (STFEs) independently complete spatio-temporal feature extraction, and then concatenate the obtained 32-dimensional feature vectors along the channel dimension to form a 128-dimensional fused representation, which is then fed into a classifier to output binary classification results of falls versus activities of daily living. This design paradigm of parallel branch concatenation and fusion has been validated in the field of gait activity recognition [43].

#### 3.4.1. Input Layer

The input of the model is four sensor data streams preprocessed by the Madgwick algorithm: the body acceleration $X_{ba}\in R^{3\times 87\times 1}$ and body angular velocity $X_{bg}\in R^{3\times 87\times 1}$ in the body frame, as well as the global acceleration $X_{ga}\in R^{3\times 87\times 1}$ and global angular velocity $X_{gg}\in R^{3\times 87\times 1}$ in the global frame. The first dimension represents the number of triaxial (*x*, *y*, *z*) channels, the second dimension T=87 is the time step (corresponding to 87 sampling points of an 875 ms time window at a 100 Hz sampling rate), and the third dimension is 1, representing single-channel features.

#### 3.4.2. Spatio-Temporal Feature Extraction Branch

Each input signal is processed by an independent STFE, and the four STFEs have completely identical structures but independent parameters. As shown in Figure 4, each STFE is composed of two DS-SE modules, a max-pooling layer, and a DS-TCN module in series, extracting motion features step by step in the time dimension.

1. DS-SE Module

The DS-SE module combines depthwise separable convolution with the channel attention mechanism. In the feature extraction stage, the number of parameters of standard convolution is proportional to $C_{in}\times C_{out}\times k$, resulting in high computational cost. In this paper, it is replaced with depthwise separable convolution, which decomposes the convolution operation into depthwise convolution and pointwise convolution. The number of parameters is compressed from $C_{in}\times C_{out}\times k$ to $C_{in}\times k+C_{in}\times C_{out}$.

For DS-SE1 ($C_{in}=3,C_{out}=16,k=9$) and DS-SE2 ($C_{in}=16,C_{out}=32,k=7$), the compression ratios reach approximately 5.76 and 5.74 times respectively, significantly reducing the spatial complexity of the model. Meanwhile, a larger convolution kernel size helps to expand the receptive field and enhance the ability to capture long-term temporal dependencies.

The forward process of the DS-SE module is as follows: The input *X* first undergoes depthwise separable convolution, batch normalization, and SiLU activation to obtain the intermediate feature *H*. Then, the SE attention module takes *H* as input, compresses *H* into a channel descriptor *z* along the time dimension through global average pooling, and then learns the importance weight *s* of each channel through a two-layer fully connected network (activated by ReLU in the middle and Sigmoid function at the end, with a channel reduction ratio r=6). Finally, *s* is multiplied element-wise with *H* to achieve adaptive calibration of feature channels. The complete process can be expressed as:

(12) $$H=\mathrm{SiLU}\left(\mathrm{BN}\left(\mathrm{DSConv}(X)\right)\right)$$

(13) $$z=\mathrm{GAP}\left(H\right),\;s=\sigma \left(W_{2}\cdot \mathrm{ReLU}\left(W_{1}\cdot z\right)\right)$$

(14) $$\hat{X}=s⊗H$$

where $W_{1}\in R^{\lfloor C/r\rfloor \times C}$ and $W_{2}\in R^{C\times \lfloor C/r\rfloor }$ are the weight matrices of the two fully connected layers in the SE module, respectively, and ⊗ denotes element-wise multiplication. It is worth emphasizing that the weight calculation and scaling operations of the SE module are both applied to the features after batch normalization and SiLU activation.

During the forward propagation of the STFE, the input signal $X\in R^{3\times 87\times 1}$ first passes through the first DS-SE module (DS-SE_1_, input channel 3, output channel $C_{1}=16$, convolution kernel size $k_{1}=9$) to extract coarse-grained motion patterns in a large receptive field. Then, downsampling is performed through a max-pooling layer with a stride of 2. Next, the second DS-SE module (DS-SE_2_, input channel 16, output channel $C_{2}=32$, convolution kernel size $k_{2}=7$) further extracts fine-grained body temporal features. The above process can be expressed as:

(15) $$H_{1}={\mathrm{DS}\text{-}\mathrm{SE}}_{1}\left(X\right),\;H_{1}\in R^{16\times 87\times 1}$$

(16) $$H_{2}={\mathrm{DS}\text{-}\mathrm{SE}}_{2}\left(\mathrm{MaxPool}\left(H_{1}\right)\right),\;H_{2}\in R^{32\times 43\times 1}$$

2. DS-TCN Module

After the CNN feature extraction is completed, this paper introduces the DS-TCN module to model the global temporal dynamics before falls. Compared with the standard causal convolution used in the original TCN [44], this paper replaces it with Causal Depthwise Separable Convolution (CDSC), which further compresses parameters while ensuring causal constraints.

Causality is achieved by left-side zero-padding: for a convolution with dilation factor *d* and kernel size *k*, the padding amount is (k−1)×d, and the equivalent output on the right side is cropped after convolution, ensuring that the output at time *t* only depends on the historical information at and before *t*, avoiding future information leakage.

3. LTB module

As shown in Figure 4, the DS-TCN is composed of two Lightweight Temporal Blocks (LTBs) in series. Each LTB contains two layers of causal depthwise separable convolution, followed by batch normalization, SiLU activation, and Dropout (p=0.08) sequentially, and the residual connection is used to alleviate the gradient vanishing problem. After residual addition, SiLU activation is performed again for output. The dilation factor of the *i*-th LTB is

(17) $$d_{i}=2^{i}\;\left(i=0,1\right)$$

with a convolution kernel size k=3. When the input and output channels are inconsistent, the residual path performs dimension matching through 1×1 convolution. In the experimental configuration of this paper, the number of channels in each layer is set to 32, and the residual path directly performs identity mapping. The calculation process of LTB can be expressed as:

(18) $$O_{i}=\mathrm{Drop}\left(\mathrm{SiLU}\left(\mathrm{BN}\left({\mathrm{CDSC}}_{i,2}\left(\mathrm{Drop}\left(\mathrm{SiLU}\left(\mathrm{BN}\left({\mathrm{CDSC}}_{i,1}\left(Y_{i-1}\right)\right)\right)\right)\right)\right)\right)\right)$$

(19) $$Y_{i}=\mathrm{SiLU}\left(O_{i}+\mathrm{Res}\left(Y_{i-1}\right)\right)$$

where Res(·) denotes the identity mapping or 1×1 convolution. The dilation factors of the two LTB layers are 1 and 2, respectively. With a kernel size k=3, the receptive field of the DS-TCN is $1+2\times \left(k-1\right)\times \left(2^{L}-1\right)=13$ time steps (where L=2 is the number of layers), corresponding to approximately 26 original sampling points before MaxPool downsampling, which is sufficient to cover the key motion information in the pre-impact phase before a fall.

After the output of DS-TCN is processed by group normalization and global average pooling, the temporal features of each branch are compressed into a 32-dimensional vector $f_{i}\in R^{32}$. Group normalization is adopted here instead of batch normalization because, in small-batch training scenarios, group normalization does not rely on batch statistics and achieves more stable normalization performance.

#### 3.4.3. Feature Fusion Layer

The output feature vectors of the four parallel branches are concatenated along the channel dimension to obtain a 128-dimensional fused feature vector:

(20) $$f=\mathrm{Concat}\left(f_{1},f_{2},f_{3},f_{4}\right)\in R^{128}$$

This concatenation operation enables the model to simultaneously utilize complementary information from different coordinate systems and different motion measurements.

#### 3.4.4. Classification Layer

The fused feature vector is directly fed into a two-layer fully connected classifier to complete fall detection. The first fully connected layer maps the dimension from 128 to 48, followed by batch normalization, SiLU activation, and Dropout (p=0.30) for regularization sequentially. The second fully connected layer maps the 48-dimensional feature to a 2-dimensional output, and the posterior probability distribution of falls and activities of daily living is obtained after normalization by the Softmax function. The complete classification path is

(21) $$\hat{y}=\mathrm{Softmax}\left(W_{4}\cdot \mathrm{Drop}\left(\mathrm{SiLU}\left(\mathrm{BN}\left(W_{3}\cdot f+b_{1}\right)\right)\right)+b_{2}\right)$$

where $f\in R^{128}$ is the feature vector after concatenation of the four branches; $W_{3}\in R^{48\times 128}$ and $b_{1}\in R^{48}$ are the weight matrix and bias vector of the first fully connected layer; $W_{4}\in R^{2\times 48}$ and $b_{2}\in R^{2}$ are the weight matrix and bias vector of the second fully connected layer.

If the predicted probability of the fall category exceeds the threshold of 0.5, it is determined as a fall event; otherwise, it is determined as an activity of daily living.

## 4. Results and Discussion

### 4.1. Experimental Setup

#### 4.1.1. Experimental Environment and Training Parameters

The proposed model is implemented using PyTorch 2.8.0 and Python 3.10.19. All experiments are conducted under a unified hardware environment to ensure reproducibility. The training and testing platform is equipped with an Intel (R) Core (TM) Ultra 7 255HX processor, 16 GB RAM, and an NVIDIA GeForce RTX 5060 Laptop GPU. To guarantee objective and fair performance evaluation, fixed hyperparameters are used across all experiments. The detailed configuration is as follows:

- Optimizer: AdamW with an initial learning rate of 0.001 and a weight decay of $8\times 10^{-5}$ to mitigate overfitting;
- Learning Rate Scheduler: Cosine Annealing with Warm Restarts ($T_{0}=15$ epochs, $T_{\mathrm{mult}}=2$, $\eta_{\min}=1\times 10^{-6}$), which dynamically adjusts the learning rate during training to improve convergence stability;
- Loss Function: Class-weighted cross-entropy with label smoothing (smoothing coefficient = 0.01) to address class imbalance and enhance generalization;
- Training Configuration: Batch size = 64, number of training epochs = 105, gradient clipping with a maximum $\ell_{2}$ norm of 0.8 to stabilize training, all experiments fix the random seed to 42 (applied to NumPy, PyTorch, and CUDA) to ensure full reproducibility;
- Data Isolation Strategy: The test set is kept fully isolated during training and is not involved in any form of parameter update or tuning. Only one independent inference run is performed after each fold of training to strictly avoid data leakage.

#### 4.1.2. Dataset

During the training and testing of the model, the KFall dataset [40] is used. The KFall dataset collects triaxial acceleration, triaxial angular velocity, and triaxial Euler angle data using a 9-axis inertial sensor at the waist with a sampling rate of 100 Hz. Based on synchronized high-definition videos, the KFall dataset annotates the fall start time and fall impact time, which greatly facilitates pre-impact fall detection. The dataset contains 5075 samples from 32 subjects, including 2729 samples covering 21 types of ADL and 2346 samples covering 15 types of falls, covering most typical real-world scenarios.

In the preprocessing stage, each fall trial is truncated at 250 ms before the impact time, and then 875 ms time windows are extracted via a reverse sliding window approach. Four fall trials were excluded because the impact occurred less than 1 s after the start of recording, resulting in insufficient valid data after truncation to form a full window. Finally, a total of 2342 fall trials were included in training and evaluation.

#### 4.1.3. Data Partition and Data Augmentation

Although the number of fall samples and ADL samples is roughly equivalent in the raw data, since ADL usually lasts for a long time (up to tens of seconds) while the fall phase is usually less than 1 s [21,45], the actual number of fall samples obtained after sliding window sampling is far less than that of ADL samples. To solve the sample imbalance problem caused by this, this study introduces data augmentation techniques for fall samples in the training set. Considering the particularity of fall events, three physically meaningful augmentation methods are adopted: jitter to simulate noise in sensor measurements; scaling to simulate the fall process of people with different body types; time warping to simulate individual differences in fall speed.

Since the dataset is measured with subjects as the basic unit, if the training set and test set are divided by random allocation, the model will overestimate the evaluation metrics due to learning individual differences in subjects. Therefore, this paper divides the training set and test set based on subject-wise grouping, strictly ensuring that all segments of the same subject do not appear in both the training set and test set, so as to truly reflect the generalization ability of the model to unseen subjects.

#### 4.1.4. Evaluation Metrics and Assessment Strategy

To objectively compare the performance differences of the models, this paper adopts commonly used evaluation metrics, including Accuracy (Acc), Recall (Rec), Specificity (Spe), Precision (Prec), and F1-Score (F1):

(22) $$\mathrm{Acc}=\frac{\mathrm{TP}+\mathrm{TN}}{\mathrm{TP}+\mathrm{TN}+\mathrm{FP}+\mathrm{FN}},$$

(23) $$\mathrm{Rec}=\frac{\mathrm{TP}}{\mathrm{TP}+\mathrm{FN}},$$

(24) $$\mathrm{Spe}=\frac{\mathrm{TN}}{\mathrm{TN}+\mathrm{FP}},$$

(25) $$\mathrm{Prec}=\frac{\mathrm{TP}}{\mathrm{TP}+\mathrm{FP}},$$

(26) $$F1=2\times \frac{\mathrm{Prec}\times \mathrm{Rec}}{\mathrm{Prec}+\mathrm{Rec}}.$$

A fall event consists of multiple consecutive window fragments, and an effective alarm can be triggered by correctly identifying just one fragment. Therefore, measuring model performance at the window-level can be misleading [22]. To address this, this study employs the trial-wise max-voting strategy to calculate the above metrics: for all sliding windows within the same trial, the highest predicted probability is taken as the final score for that trial. If this score is not lower than the decision threshold of 0.5, the event is determined as a fall. This strategy is equivalent to the real-time detection logic of “triggering an alarm when the single-window probability exceeds the threshold” in actual deployment scenarios, ensuring that the evaluation results have practical deployment significance. At the same time, to ensure comparability with existing literature, this paper also introduces standard window-level evaluation as an auxiliary evaluation strategy.

### 4.2. Model Performance Comparison

To verify the detection performance of MadgwickFall-Net, this study conducts comparative experiments with several other deep learning models for pre-impact fall detection.

To ensure the fairness of the comparison, all models are evaluated using 5-fold GroupKFold cross-validation on the same KFall dataset. The data preprocessing, window length, and calculation method of evaluation indicators are completely consistent with MadgwickFall-Net, while the input features of each comparative model remain consistent with their original papers. The comparison results using the trial-level Max strategy are shown in Table 1.

Experimental results show that MadgwickFall-Net achieves the best performance in four indicators: F1-Score, Precision, Specificity, and Accuracy, with an F1-Score of 0.9824, Precision of 0.9773, Specificity of 0.9801, and Accuracy of 0.9836. In terms of Recall, MadgwickFall-Net scores 0.9876, which is only 0.0012 lower than Turetta’s CNN (0.9888), the model with the highest Recall. However, Turetta’s CNN achieves a Precision of only 0.9577, which is significantly lower than MadgwickFall-Net’s 0.9773. This indicates that MadgwickFall-Net achieves a better Precision–Recall balance while maintaining an extremely high Recall. In addition, MadgwickFall-Net has only 59.7 KB parameters, the smallest among all comparison models, which is approximately 2.2% of ConvLSTM (2742.5 KB), the model with the largest parameters, showing significant lightweight advantages.

To verify the statistical reliability of the above performance improvements, this study conducted a paired Wilcoxon signed-rank test (one-tailed, α=0.05) on the 5-fold F1-Score results of MadgwickFall-Net and all baseline models. The results show that MadgwickFall-Net achieved statistical significance (p=0.031) in 4 out of 5 comparisons. In the comparison with lwRPPC-TCN, MadgwickFall-Net led with a 4-0-1 win-tie-loss record, with a Wilcoxon p=0.063, which did not reach the significance level but remained close to the boundary. To further verify the reliability of the comparison with lwRPPC-TCN, a paired *t*-test was performed: the results show that the p-values of all 5 comparisons were no more than 0.024, including the comparison with lwRPPC-TCN (p=0.014). This indicates that MadgwickFall-Net achieved statistically significant performance improvements over all baseline models when considering the magnitude of differences. In addition, Cohen’s *d* ranged from 1.28 to 4.45, all far exceeding the large effect size threshold of 0.8. Combining the two test methods and effect size analysis, the performance improvements of MadgwickFall-Net over all baseline models are both statistically significant and practically meaningful, eliminating the influence of random fluctuations.

Further analysis of the causes of performance differences among the above models reveals that the choice of input feature design is one of the key influencing factors. CNN Dense uses only triaxial acceleration and its derived SVM and tilt angles as inputs; ConvLSTM and lwRPPC-TCN use raw acceleration and angular velocity; PreFallKD and Turetta’s CNN additionally introduce Euler angles calculated by magnetometer fusion based on acceleration and angular velocity. From the comparison results, the detection performance of each model generally shows an upward trend as input features are gradually enriched. However, introducing magnetometer-based Euler angles not only increases hardware cost and is susceptible to magnetic field interference, but also its reliance on a specific fusion process can lead to training-deployment mismatch problems in real-world scenarios; models using only raw inertial signals fail to fully exploit attitude kinematic features. MadgwickFall-Net relies only on acceleration and angular velocity signals from common sensors, achieving more comprehensive feature extraction without additional magnetometer-dependent inputs, which is the fundamental reason for its significant advantages in precision and specificity.

To further ensure comparability with existing literature, Table 2 presents the experimental results of all models under the standard window-level evaluation strategy. Compared with the trial-level results, the F1-Score, precision, and recall of all models decreased under window-level evaluation, which stems from the essential difference in statistical granularity between the two strategies: trial-level evaluation is conducted on a per-trial basis: for recall, a fall trial is considered detected as long as any window’s probability exceeds the threshold, without requiring all windows to be correctly classified; for precision, regardless of how many windows are misclassified in an ADL trial, it is counted as a single false positive. Therefore, the trial-level strategy is more lenient for both recall and precision. In contrast, window-level evaluation requires independent judgment for each window, with both missed detections and false positives accumulated on a per-window basis, leading to a synchronous decrease in both metrics and a corresponding drop in F1-Score. Nevertheless, MadgwickFall-Net still leads in four metrics: F1-Score, precision, specificity, and accuracy under window-level evaluation, verifying the robustness of the model’s performance.

This paper conducted a per-activity false positive analysis on all 2729 ADL trials in the 5-fold test sets, and the results are shown in Table 3. The overall false positive rate is 2.02%. Static and low-dynamic activities (T01, T03–T04, T06–T09, T11–T14) exhibit extremely low false positive rates, with activities such as sitting, lying down, static lying, jumping, and walking downstairs achieving a 0.00% false positive rate, indicating the model’s high discrimination ability for these activities. False positives are mainly concentrated in two types of activities: falling while attempting to rise (T15, 14.65%) and stumbling while walking (T10, 8.18%). The former produces impactive acceleration features highly similar to the initial stage of a fall due to rapid forward trunk flexion followed by sudden loss of support and rebound acceleration; the latter involves abrupt forward trunk tilt and sudden center-of-gravity shift at the moment of stumbling, whose attitude change amplitude largely overlaps with the early kinematic patterns of real falls. These two activities, whose kinematic features are the most similar to falls, represent inherent challenges in pre-impact fall detection tasks.

In practical deployment, the decision threshold can be flexibly adjusted according to the risk preference of the application scenario: for low-risk scenarios with high tolerance for false positives, the threshold can be appropriately increased to reduce false positives caused by such activities; for high-risk scenarios where the cost of missed detection is high (e.g., elderly people living alone, bedridden patients), a lower threshold should be maintained to prioritize recall.

### 4.3. Ablation Study

#### 4.3.1. Coordinate System Ablation Experiment

To quantitatively evaluate the specific contribution of the Madgwick algorithm and dual-frame parallel modeling strategy to detection performance, this study designs an ablation experiment targeting the model input. The experiment includes three configurations:

1. Four-branch complete model (i.e., MadgwickFall-Net proposed in this paper, fusing body and global features).
2. Global frame only (only inputting globally transformed acceleration and angular velocity via Madgwick algorithm).
3. Body frame only (without Madgwick, only inputting body frame acceleration and angular velocity).

To ensure fairness of comparison, after removing unnecessary parallel branches, Experiments 2 and 3 retain the same CNN + DS-TCN backbone feature extraction structure and fully connected layer configuration as the complete model. The statistical results of 5-fold cross-validation are shown in Table 4.

**Figure 5:**
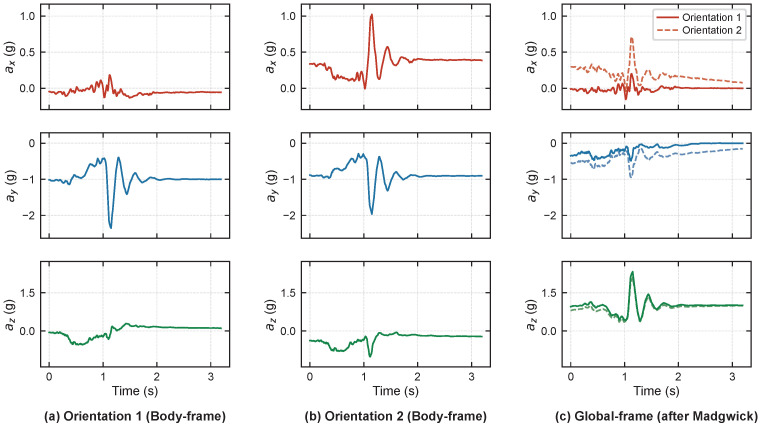
Effect of sensor wearing orientation on body frame accelerometer signals and its correction by the Madgwick algorithm (Subject SA06, forward fall). Rows show $a_{x}$, $a_{y}$, and $a_{z}$ respectively, represented by red, blue, and green lines. (**a**) Body frame signals at the original wearing orientation. (**b**) Body frame signals after a simulated placement offset (Δroll=20, Δpitch=15, Δyaw=30). (**c**) Signals from both orientations transformed to the global frame via the Madgwick algorithm, demonstrating alignment of the fall kinematics across orientations; solid lines denote Orientation 1 and dashed lines denote Orientation 2.

To intuitively demonstrate the impact of coordinate transformation on signal characterization, taking the acceleration signal of subject SA06 performing a forward fall as an example, Figure 5 compares the signal differences before and after changes in wearing orientation, as well as the correction effect of the Madgwick algorithm. As shown in Figure 5, even with only a total deviation of approximately 35° in the wearing orientation (Δroll = 20°, Δpitch = 15°, Δyaw = 30°), the triaxial acceleration waveforms in the body frame change significantly. In contrast, after transforming to the global frame via the Madgwick algorithm, the waveforms under the two wearing orientations converge substantially, and the key motion characteristics of the fall event are effectively aligned. This phenomenon intuitively verifies the effectiveness of coordinate transformation in eliminating orientation dependency.

Experimental results show that the global-frame-only model (Exp. 2) achieves an F1-Score of 0.9723 and an Accuracy of 0.9742, significantly outperforming the body-frame-only model (Exp. 3) with 0.9660 and 0.9682, respectively. The four-branch full model (Exp. 1) yields the best performance across all evaluation metrics, with an F1-Score of 0.9824, an Accuracy of 0.9836, a Precision of 0.9773, and a Specificity of 0.9801. Compared with the global-frame-only model, the four-branch full model improves the F1-Score and Accuracy by 0.0101 and 0.0094, respectively.

The above performance differences can be explained from two levels. At the coordinate transformation level, body frame signals are strongly affected by the sensor wearing orientation. The same fall motion produces completely different acceleration components at different wearing orientation, making it difficult for the model to learn rotation-invariant fall features. After the Madgwick algorithm transforms body measurements into the global frame, the macroscopic motion characteristics of the human body’s center of mass relative to the direction of gravity during the fall are uniformly characterized, enabling the neural network to more accurately capture the kinematic abrupt change mode at the moment of the fall. This is why the global-frame-only model outperforms the body-frame-only model in all metrics. At the feature complementarity level, the global frame excels in depicting the overall motion trajectory and posture evolution trend of the human body’s center of mass, while the body frame additionally retains high-frequency limb compensatory actions and microscopic angular velocity fluctuations in the early stage of imbalance. These transient features are key clues to distinguish falls from intense daily activities. The complete model simultaneously leverages the complementarity of the two types of information through the four-branch parallel structure, further improving the F1-Score by 0.0101 compared with the global-frame-only model.

**Figure 6:**
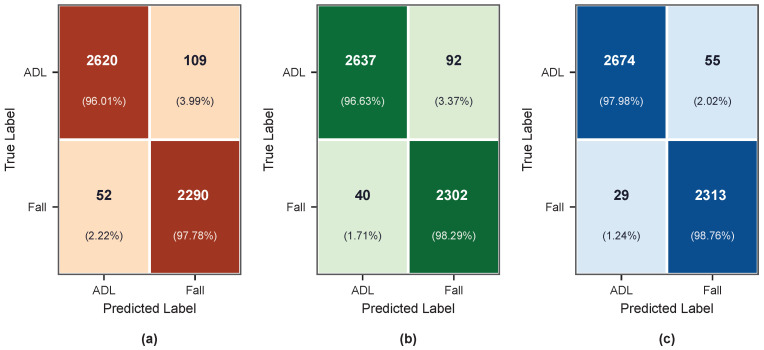
Confusion matrices of averaged 5-fold cross-validation results for the coordinate system ablation study (trial-wise max-voting strategy, threshold = 0.5), illustrating the progressive reduction in false positives as coordinate system information is enriched. (**a**) Body frame only (without Madgwick). (**b**) Global frame only (Madgwick output only). (**c**) MadgwickFall-Net (four-branch full model, proposed).

Further analysis of the confusion matrices in Figure 6 reveals that with the gradual enrichment of coordinate system information, the number of false positives decreases from 109 cases to 92 cases, and finally converges to 55 cases, a reduction of 49.5%; meanwhile, the number of false negatives remains consistently low across all three configurations. This study adopts the trial-level Max strategy for evaluation, under which an alert is triggered as long as at least one window in a trial is correctly identified. This makes recall insensitive to coordinate system design, allowing differences in false positive control capabilities to be more fully reflected. The above changes in the confusion matrices demonstrate that the fusion of dual-coordinate features effectively enhances the model’s ability to recognize complex activities of daily living, which is the fundamental reason for the significant reduction in the false positive rate.

#### 4.3.2. Window Length Assessment

**Figure 7:**
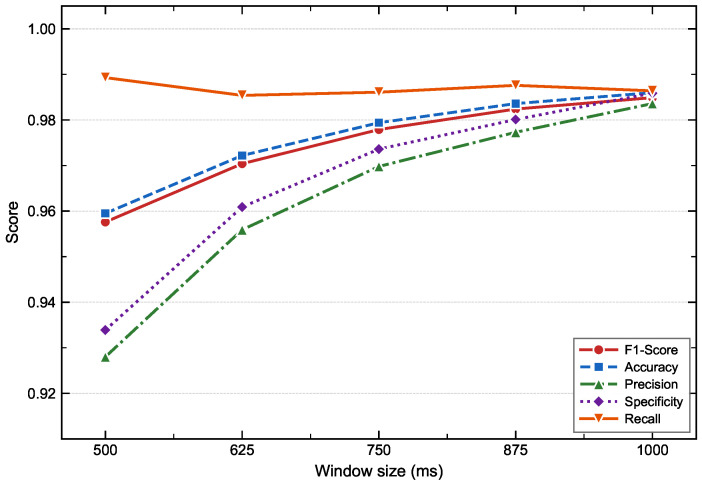
Effect of sliding window size on fall detection performance under 5-fold group cross-validation. Data points represent mean scores of five evaluation metrics across five window sizes (500–1000 ms, step 125 ms).

The size of the sliding window directly determines the temporal receptive field of the model. To investigate the specific impact of temporal context information on fall detection performance, this study sets five different window configurations in the range of 500 ms to 1000 ms with a step interval of 125 ms, and conducts a systematic comparative ablation experiment for each configuration. During the experiment, except for the input window length, the network architecture and other hyperparameters are strictly kept consistent. The 5-fold cross-validation results under each configuration are shown in Figure 7.

Experimental results show that as the window length increases from 500 ms to 1000 ms, the F1-Score, Accuracy, Precision, and Specificity all exhibit a continuous monotonic upward trend, improving from 0.9576, 0.9595, 0.9280, and 0.9339 to 0.9849, 0.9860, 0.9836, and 0.9859, respectively. In contrast, Recall fluctuates within a narrow range at a high level, varying from 0.9885 to 0.9900 without showing a significant monotonic trend. This indicates that within the tested range, window size has a relatively limited impact on the model’s ability to capture fall events.

From the perspective of the marginal effect of performance gain, the improvement in each metric shows a clear diminishing return as the window size increases. Taking the F1-Score as an example, the gain from 500 ms to 625 ms is 0.0128, from 625 ms to 750 ms, it is 0.0075, from 750 ms to 875 ms, it narrows to 0.0045, and from 875 ms to 1000 ms, it is only 0.0025, less than one-third of the average gain of the first three intervals. Accuracy, Precision, and Specificity exhibit the same diminishing trend. This indicates that the model has already fully extracted the temporal features of fall movements at 875 ms, and the additional discriminative information brought by further expanding the window is extremely limited.

At the same time, the window size itself constitutes a lower bound on the system response latency: the model must wait for the complete window of data to accumulate before completing inference, so the larger the window, the longer the minimum system response time. In fall detection scenarios, response latency is directly related to injury prevention effectiveness, and trading a small precision gain for additional system latency is not justified. Combining the diminishing marginal returns of performance gains and the latency constraints of real-time deployment, this study uniformly sets the sliding window size to 875 ms.

#### 4.3.3. Madgwick Algorithm β Parameter Ablation Experiment

**Figure 8:**
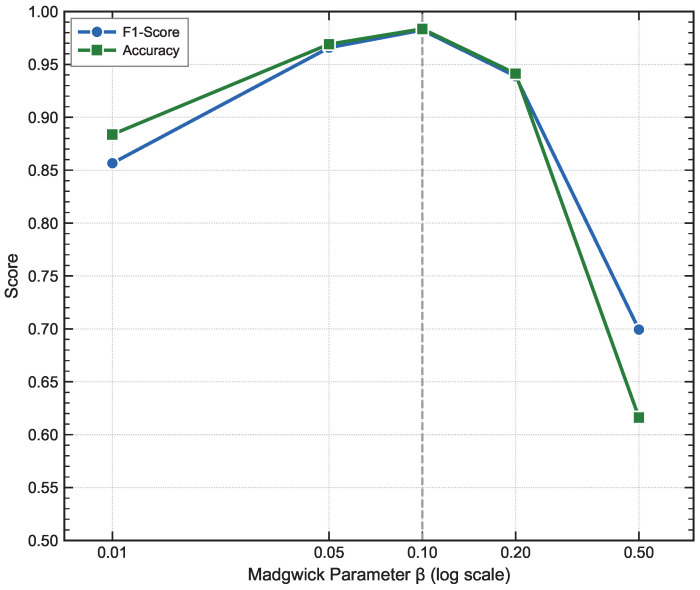
Ablation study on the Madgwick algorithm parameter β. F1-Score and Accuracy are reported as mean over 5-fold cross-validation. The x-axis is displayed on a logarithmic scale to reflect the proportional spacing of the tested values (β∈{0.01,0.05,0.1,0.2,0.5}). The dashed vertical line indicates the selected value β=0.1.

The β parameter in the Madgwick algorithm controls the correction weight of the accelerometer on attitude estimation: when β is too small, attitude fusion mainly relies on gyroscope integration, which is prone to attitude drift; when β is too large, accelerometer noise is excessively introduced, leading to distortion in the global frame estimation [23]. To determine the optimal β value, this study conducts a 5-fold cross-validation ablation experiment with β∈{0.01,0.05,0.1,0.2,0.5}, and the results are shown in Figure 8.

As shown in Figure 8, the trends of F1-Score and Accuracy are highly consistent, both exhibiting an inverted U-shaped curve. When β=0.01, both metrics are significantly low, indicating that excessive reliance on gyroscope integration leads to attitude drift, which severely degrades detection performance. As β increases, performance improves rapidly, reaching its optimum at β=0.1. Further increasing β results in performance degradation, showing that excessive introduction of accelerometer noise deteriorates the quality of coordinate transformation. Overall, β=0.1 achieves the optimal balance between dynamic tracking response and noise suppression, and this value is adopted as the final setting in this paper.

### 4.4. Timing Validation and Deployment

#### 4.4.1. Pre-Impact Lead-Time Validation

It should be noted that the 250 ms data cutoff set during training is not a guaranteed warning lead time in practical deployment, but a training boundary constraint determined based on the average deployment time of approximately 200 ms for wearable airbags. Its purpose is to ensure that the model only learns motion patterns before impact and reserves computational margin for inference. The actual warning lead time of the model depends on the recognizability of fall motion characteristics in specific trials and needs to be quantified through sliding-window inference experiments.

To this end, this paper conducts rolling inference experiments on all fall trials in the five-fold cross-validation test sets, to directly measure the model’s pre-impact lead time under real-world deployment scenarios. This experiment adopts the forward sliding window strategy that is fully consistent with actual deployment to simulate the inference behavior in continuous real-time monitoring scenarios. For each fall trial, the complete signal sequence is processed with the same 8 Hz Butterworth low-pass filter and Madgwick attitude estimation as in the training phase. Subsequently, an 875 ms sliding window is advanced from the start of the recording to the moment before the labeled impact frame, with a step size of 340 ms (consistent with the training step size). For each window, the model outputs a fall probability; the first time it exceeds the threshold of 0.5 is recorded as the alarm time $t_{\mathrm{alarm}}$. The pre-impact lead time is defined as the difference between the impact time $t_{\mathrm{impact}}$ and the alarm time:

(27) $$\Delta t=t_{\mathrm{impact}}-t_{\mathrm{alarm}}$$

**Figure 9:**
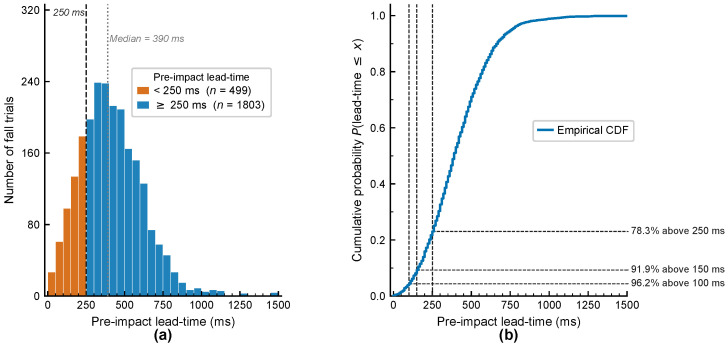
Pre-impact lead-time distribution of MadgwickFall-Net evaluated via rolling inference on 2342 fall trials across the 5-fold test sets. (**a**) Frequency histogram (bin width = 50 ms); bars colored orange (<250 ms) or blue (≥250 ms). (**b**) Empirical cumulative distribution function (CDF); horizontal reference lines indicate the proportions of trials exceeding 100, 150, and 250 ms.

This study conducted rolling inference evaluation on all 2342 fall trials in the five-fold test set. MadgwickFall-Net successfully triggered an alarm before impact in 2302 trials, yielding an overall detection rate of 98.3%. Among these 2302 detected trials, the median pre-impact lead time was 390 ms, with a mean of 410 ms. As shown in Figure 9, in terms of compliance rate, 78.3% of detected trials achieved a lead time of no less than 250 ms, 91.9% no less than 150 ms, and 96.2% no less than 100 ms. Taking the average deployment time of 200 ms for wearable airbags as a reference, 86.1% of detected trials had a lead time exceeding 200 ms, indicating that the system meets the time requirement to trigger and fully deploy the airbag in the vast majority of detection scenarios. For trials with a lead time below 200 ms, although insufficient to fully deploy the airbag, they can still trigger faster-response auxiliary protection devices, thus still providing practical protective value in real-world deployment.

The remaining 40 trials did not trigger an alarm before impact, constituting missed detections in this experiment, with a miss rate of 1.7%. Analysis of the model outputs for these missed trials reveals that 29 out of 40 cases (72.5%) had a maximum prediction probability below 0.30, indicating that the motion patterns in these trials were inherently dissimilar to the fall modes in the training data, rather than being caused by improper threshold settings. Only 5 cases had a maximum prediction probability of 0.40 or higher, suggesting potential for improving detection rate by lowering the threshold.

The sliding-window inference in this experiment further builds on the main experiment (Section 4.2) by quantifying the pre-impact detection lead time to validate and supplement the practical performance of the model from the perspective of sequential early warning.

#### 4.4.2. Deployment Consistency Validation: Forward vs. Reverse Sliding Window

As described in Section 3.2, the reverse sliding window strategy is only used for offline dataset construction during the training phase, while the real-time inference system adopts a forward-sliding buffer for continuous processing. To quantitatively evaluate the actual impact of this train-deployment discrepancy on model performance, this study designs a subject-independent forward sliding window mitigation experiment: the reverse sliding window strategy for fall data is replaced with the same forward sliding window as used for ADL data, and the rolling inference scheme identical to that in Section 4.4.1 is adopted to quantify the pre-impact lead time, with all other hyperparameters, network architecture, and training configurations kept unchanged. The comparative results of 5-fold cross-validation are shown in Table 5.

Experimental results show that the trial-level F1-Score of the Forward (Fall) + Forward (ADL) configuration is 0.8754, with a Recall of 0.7954, which decreases by 0.1070 and 0.1922, respectively, compared to the Reverse (Fall) + Forward (ADL) configuration. Meanwhile, Precision and Specificity are nearly equivalent between the two configurations, indicating that the root cause of performance degradation lies in the decline in fall event detection capability rather than an increase in false positive rate. This discrepancy arises because the forward sliding window strategy discards the terminal data segment closest to the cutoff point, which precisely contains the most discriminative motion features such as the rapid sinking of the trunk’s center of gravity and abrupt acceleration changes. By anchoring the end of each training window to the cutoff point, the reverse strategy maximally preserves the motion patterns in this critical region, thereby endowing the model with stronger fall detection capability.

From the rolling inference experiments, the median lead time of the two configurations differs by only 10 ms. The overall detection rate of the Forward (Fall) + Forward (ADL) configuration is 97.4%, which is lower than the 98.3% of the Reverse (Fall) + Forward (ADL) configuration, consistent with the sharp drop in trial-level Recall from 0.9876 to 0.7954. The above results collectively confirm from an experimental perspective that the reverse sliding window strategy does not introduce systematic train-deployment mismatches; its value lies in effectively improving the model’s ability to detect fall events through optimized offline dataset construction.

#### 4.4.3. Embedded Platform Deployment

Focusing on the attitude convergence characteristics of the Madgwick algorithm, this paper targets the STM32H7 series platform with a main frequency of 480 MHz. Its timing behavior is simulated on a computer through instruction-level static analysis, and quantitative warm-up experiments are conducted based on the standing still task (D01, lasting 30 s) of 32 subjects in the KFall dataset. Under 9 initial wearing bias configurations (Δroll/Δpitch/Δyaw non-uniformly sampled from 0° to 90°, covering single-axis and multi-axis combined biases), the static signals are processed frame-by-frame following the exact same Madgwick call logic as in the training code. Convergence is judged when the angular deviation of the global acceleration from the vertical direction remains below 5° for 0.5 consecutive seconds. The experimental results show that all 32 subjects achieved convergence within 15.33 s under all bias configurations, with the 95th percentile convergence time being 15.0 s. Considering uncertainties such as individual differences and extreme wearing angles in real deployment, an additional safety margin of approximately 5 s is reserved on this basis, setting the warm-up time to 20 s during actual deployment.

In addition, this paper estimates the computational overhead of the preprocessing pipeline through instruction-level static analysis. Based on the typical instruction latency of the FPU on the STM32H7 hardware, the two steps of Butterworth low-pass filtering and Madgwick attitude estimation are counted item by item, and it is estimated that each frame requires approximately 360 and 560 clock cycles, respectively. After a 20-second warm-up, simulating a complete inference window (87 frames), the two steps take a combined total of approximately 166.75 µs, making the preprocessing overhead negligible.

To verify the deployability of MadgwickFall-Net on resource-constrained embedded platforms, this paper performs simulation benchmark testing of the model using ST Edge AI Developer Cloud. The trained model weights are saved in FP16 format (size: 59.7 KB) and converted to FP32 during export to meet the platform’s ONNX import requirements. Three STM32 development boards are selected as simulation targets, and tests are executed with the balanced optimization strategy to record Flash usage, RAM usage, and single inference time. The results are summarized in Table 6. The experimental results show that the memory usage is identical across all platforms: total Flash is 166.1 KB (model weights: 116.4 KB; runtime kernel: 49.7 KB), and total RAM is 38.2 KB (activation buffer: 21.4 KB; kernel: 16.8 KB). The inference time varies between 18.70 ms (STM32H735G-DK) and 25.75 ms (STM32H747I-DISCO), remaining at a low level overall. Taking the longest inference time of 25.75 ms as a reference, combined with the median pre-impact lead-time of 390 ms in the rolling inference experiment, a single inference accounts for only 6.6% of the available warning window, leaving ample computational margin. Combining both preprocessing and inference stages, the end-to-end computational burden is generally controllable, meeting the response time requirements of real-time early warning systems.

Based on the aforementioned deployment metrics, the practical deployment process of MadgwickFall-Net is as follows: The IMU sensor fixed on the waist continuously collects three-axis acceleration and three-axis angular velocity signals at a sampling rate of 100 Hz. After the system is first powered on or the sensor is re-worn, it is necessary to remain upright and stationary for approximately 20 s to complete the attitude convergence warm-up of the Madgwick algorithm, during which the system suspends inference. After the warm-up is completed, the on-chip firmware sequentially performs Butterworth low-pass filtering and Madgwick attitude estimation on each newly sampled point, and writes the dual-coordinate signals into a sliding buffer with a length of 87 sampling points (875 ms) in real time. Every time 34 new sampling points are accumulated (340 ms, consistent with the window step size of 60% overlap during training), MadgwickFall-Net inference is performed once with the current content of the buffer as input. Once the output fall probability exceeds the threshold of 0.5, the system immediately triggers the protection device via GPIO. Meanwhile, the trigger signal can alert surrounding personnel through a local buzzer and be pushed to the caregiver terminal via a wireless communication module. After triggering, the system enters a lockout period with a fixed duration of 5 s. Existing studies have shown that the complete fall process from imbalance to body stabilization after impact usually ends within 3 s [48], and the 5-second setting reserves sufficient margin for residual motion after impact. During the lockout period, model inference is completely suspended and the sliding buffer is cleared, while IMU data acquisition and Madgwick attitude estimation continue to run to maintain quaternion continuity, thereby avoiding re-convergence overhead after the lockout expires. After the lockout period expires, the system automatically resets to the monitoring state, the buffer is refilled from the current frame, and inference resumes after 87 sampling points are accumulated. This mechanism ensures that each fall event triggers only one alarm, avoiding redundant outputs of the same event from consecutive windows.

## 5. Conclusions

This paper proposes DualFrame-PFD, a pre-impact fall detection framework based on dual-coordinate feature fusion, and its neural network component MadgwickFall-Net. By introducing the Madgwick algorithm to transform inertial signals into the global coordinate system, we construct orientation-invariant inertial feature representations. Relying only on two universal signals (acceleration and angular velocity), the proposed framework avoids the train-deployment mismatch problem. The four-branch parallel architecture further improves the utilization efficiency of attitude kinematic information by fusing complementary features from both coordinate systems. The experimental results comprehensively verify the effectiveness of the proposed method across three key dimensions: detection accuracy, pre-impact lead time, and embedded deployability. These findings confirm that the dual-frame fusion strategy offers substantial practical value for real-world wearable fall detection systems.

This study has the following limitations: all experimental data are from simulated fall scenarios by healthy young adults, and the generalization ability of the model in the real elderly population remains to be verified; the evaluation is based on a single dataset, and cross-dataset generalization performance has not been validated. Future work will focus on collecting data from the real elderly population to improve ecological validity, exploring false alarm suppression strategies for high-dynamic activities of daily living, and further verifying the generalization ability of the model on multiple datasets.

## Figures and Tables

**Table 1:** Fall detection performance comparison on the KFall dataset (5-fold GroupKFold cross-validation, trial-level max strategy).

Model	F1	Precision	Recall	Specificity	Accuracy	Params (KB)
CNN Dense [46]	0.8839	0.8394	0.9340	0.8452	0.8863	71.6
ConvLSTM [47]	0.9621	0.9418	0.9834	0.9477	0.9641	2742.5
lwRPPC-TCN [45]	0.9700	0.9572	0.9832	0.9622	0.9719	153.9
PreFallKD [21]	0.9724	0.9611	0.9844	0.9656	0.9742	912.1
Turetta’s CNN [22]	0.9730	0.9577	**0.9888**	0.9625	0.9747	107.4
MadgwickFall-Net (Ours)	**0.9824**	**0.9773**	0.9876	**0.9801**	**0.9836**	**59.7**

Note: Bold values indicate the best performance in each column. Gray shading indicates the results of the proposed method.

**Table 2:** Fall detection performance comparison on the KFall dataset (5-fold GroupKFold cross-validation, window-level evaluation).

Model	F1	Precision	Recall	Specificity	Accuracy	Params (KB)
CNN Dense [46]	0.7620	0.7433	0.7822	0.9878	0.9790	71.6
ConvLSTM [47]	0.8519	0.8340	0.8706	0.9922	0.9870	2742.5
lwRPPC-TCN [45]	0.8596	0.8742	0.8466	0.9945	0.9882	153.9
PreFallKD [21]	0.8663	0.8761	0.8575	0.9945	0.9886	912.1
Turetta’s CNN [22]	0.8663	0.8339	**0.9015**	0.9919	0.9881	107.4
MadgwickFall-Net (Ours)	**0.8911**	**0.9042**	0.8789	**0.9958**	**0.9908**	**59.7**

Note: Bold values indicate the best performance in each column. Gray shading indicates the results of the proposed method.

**Table 3:** Per-activity false positive analysis of MadgwickFall-Net on ADL trials across 5-fold test sets (trial-wise max-voting, threshold = 0.5; activities with FP rate > 5% highlighted in bold).

Task	Activity	Total Trials	FP	FP Rate
T01, T11, T12, T17	Static/low-dynamic activities	128	0	0.00%
T03	Pick up object from floor	157	0	0.00%
T06	Walk normally with turn	151	0	0.00%
T07	Walk quickly with turn	154	0	0.00%
T08	Jog normally with turn	156	0	0.00%
T09	Jog quickly with turn	151	0	0.00%
T13	Sit down/get up chair (normal speed)	149	0	0.00%
T14	Sit down/get up chair (quick)	155	0	0.00%
T18	Sit, lie down to bed normally, and get up	152	0	0.00%
T04	Gently jump	160	1	0.62%
T16	Stand, sit on sofa (inclined), get up	152	1	0.66%
T19	Sit, lie down to bed quickly, and get up	149	2	1.34%
T36	Walk upstairs and downstairs (quick)	149	2	1.34%
T35	Walk upstairs and downstairs (normal)	137	2	1.46%
T02	Bend back/tie shoe lace	157	5	3.18%
T05	Sit to ground and get up	156	6	3.85%
**T10**	**Stumble while walking**	**159**	**13**	**8.18%**
**T15**	**Sit a moment, trying to get up, collapse into a chair**	**157**	**23**	**14.65%**
Total		2729	55	2.02%

**Table 4:** Ablation study results on coordinate system configurations (5-fold cross-validation means).

Configuration	Branches	F1-Score	Recall	Precision	Specificity	Accuracy	ΔF1
1 four-branch	4	**0.9824**	**0.9876**	**0.9773**	**0.9801**	**0.9836**	—
2 Global Frame Only	2	0.9723	0.9833	0.9617	0.9664	0.9742	↓ 0.0101
3 Body Frame Only	2	0.9660	0.9784	0.9542	0.9596	0.9682	↓ 0.0164

Note: Δ F1 denotes the F1-Score difference relative to the four-branch full model (Configuration 1); ↓ indicates performance degradation. Bold values indicate the best performance in each column.

**Table 5:** Ablation study on sliding window sampling strategies (5-fold GroupKFold cross-validation, trial-level max voting).

Strategy	F1	Rec	Prec	Spe	Acc	Median Lead Time	Detection Rate
R(F) + F(A)	**0.9824**	**0.9876**	**0.9773**	0.9801	**0.9836**	390 ms	**98.3%**
F(F) + F(A)	0.8754	0.7954	0.9741	**0.9818**	0.8957	**400 ms**	97.4%
Δ	↓ 0.1070	↓ 0.1922	↓ 0.0032	↑ 0.0017	↓ 0.0879	↑ 10 ms	↓ 0.9%

Note: R(F) = Reverse sampling for fall data; F(F) = Forward sampling for fall data; F(A) = Forward sampling for ADL data; ↑/↓ indicates improvement/degradation. Bold values indicate the best performance in each column.

**Table 6:** Simulated deployment results of MadgwickFall-Net on three STM32 Platforms (ST Edge AI Developer Cloud, FP32, Balanced Optimization).

Board	CPU Frequency (MHz)	Inference Time (ms)	Clock Cycles	Flash (KB)	RAM (KB)
STM32H735G-DK	550	18.70	10,285,888	166.1	38.2
NUCLEO-H743ZI2	480	21.47	10,306,181	166.1	38.2
STM32H747I-DISCO	480	25.75	10,299,366	166.1	38.2

## Data Availability

The data used in this study are publicly available. The KFall dataset can be accessed at https://sites.google.com/view/kfalldataset (accessed on 15 May 2026 ).
